# Establishing Minimal Clinically Important Differences for the Quality of Life Instrument in Patients With Breast Cancer QLICP-BR (V2.0) Based on Anchor-Based and Distribution-Based Methods

**DOI:** 10.3389/fonc.2022.753729

**Published:** 2022-05-02

**Authors:** Fei Li, Yuxi Liu, Chonghua Wan, Jiali Zhou, Jianfeng Tan, Huanwei Chen

**Affiliations:** ^1^ Research Center for Quality of Life and Applied Psychology, Key Laboratory for Quality of Life and Psychological Assessment and Intervention, School of Humanities and Management, Guangdong Medical University, Dongguan, China; ^2^ Central Hospital of Guangdong Nongken, The six wards of Medical Oncology, Zhanjiang, China

**Keywords:** breast cancer, quality of life, minimal clinically important difference, anchor-based method, distribution-based method

## Abstract

**Objective:**

To determine the minimal clinically important differences (MCIDs) for the breast cancer scale QLICP-BR (V2.0) among the Quality of Life Instruments system for cancer patients (QLICP), which consist of the general module of 32 items classifying into 4 domains and the specific module of 10 items.

**Methods:**

According to the scoring rule of QLICP-BR (V2.0), the scores of each domain and the overall scale were calculated. The MCIDs of this scale were established by anchor-based and distribution-based methods. The anchor method used the Q29 item in the EORTC QLQ-C30 scale as anchors and defined the treatment effectiveness of the anchor-based method using criteria A (one level improvement after treatment) and B (at least one level improvement after treatment), while methods of effect size (ES), standard error of measurement (SEM), and reliability change index (RCI) were used in distribution-based methods.

**Results:**

Using the anchor-based method, according to standard A, the MCIDs of the physical domain (PHD), psychological domain (PSD), social domain (SOD), common symptoms and side effect domain (SSD), core/general module (CGD), specific domain (SPD), and the total score (TOT) were 16.24, 11.37, 11.31, 12.07, 11.49, 10.69, and 11.23 respectively; according to standard B, the MCIDs of PHD, PSD, SOD, SSD, CGD, SPD, and TOT were 18.88, 15.14, 14.10, 14.50, 13.93, 12.17, and 14.23 respectively. In the distribution-based MCID study, when ES = 0.8, the MCID values of each domain and the total score of the scale were 9.14, 10.34, 8.34, 10.54, 6.79, 9.73, and 6.96 respectively. The MCIDs calculated when a SEM of 1.96 was used as the intermediary index were 8.38, 11.04, 8.67, 10.00, 7.44, 9.83, and 7.81. The MCIDs calculated when a RCI of 1.96 was used as the intermediary index were 11.84, 15.61, 12.27, 14.14, 10.52, 13.90, and 11.05. Additionally, the MCID value calculated by the two standards of the anchor method was similar to 0.8 ES, 1.96 SEM, and 1.96 RCI.

**Conclusion:**

Using the anchor-based method, 0.8ES, 1.96SEM, and 1.96RCI have a better effect on the minimal clinically important difference of breast cancer scale and were recommended to be the preferred methods for establishing MCID.

## Introduction

Breast cancer is one of the most common malignant tumors in women and a leading cause of cancer-related deaths. The age of breast cancer diagnosis is between 40 and 60 years old. Each year more than 1.7 million women are diagnosed and more than 500 000 die from breast cancer, making it the leading cause of cancer death among women globally (1, 2). There are more than 1.67 million new cases of breast cancer in women worldwide in 2012, ranking first place in the incidence of female malignant tumors (3–5). In 2018, breast cancer accounted for 11.6% of all cancer deaths worldwide (6). In China, the incidence of breast cancer has increased in recent years with the development of society and economy, changes in lifestyle, and ecological environment. From 2003 to 2008, the standardized incidence of breast cancer in Chinese women increased from 21.17/100,000 to 26.26/100,000, an increase of 17.65%. The incidence of breast cancer declined slightly in 2009, but from 2010 to 2012, it rose sharply to 30.43/100,000, an increase of 43.74% compared with 2003. The average annual rate of change in breast cancer mortality over the past 10 years was 3.87 percent (7). It can be seen from this that the incidence and mortality of breast cancer in China as a whole show a trend of a gradual increase, and the burden of disease is also increasing.

Along with the increasing number of breast cancer patients and the longer survival due to early detection programs and advancement in medical technology, accurately assessing the health-related quality of life (HRQOL) of breast cancer patients is crucial (8–10). By far, the more popular HRQOL tools for breast cancer are the European Organization for Research and Treatment (EORTC) Quality of Life Questionnaire-C30 (QLQ-C30) and the breast cancer-specific module QLQ-BR23, and the American Breast Cancer Quality of Life Measurement Scale FACT-B (10–12).

However, these scales are not fully applicable to the assessment of the quality of life of Chinese people. Therefore, Wan’s QOL team developed the Chinese instrument for the breast cancer quality of life measurement QLICP-BR (including the first (13) and second editions), which is one scale of the system of Quality of Life Instruments for cancer patients (QLICP). The second version QLICP-BR (V2.0) is composed of a general module QLICP-GM (V2.0) and a breast cancer-specific module. Among them, QLICP-GM (V2.0) includes 32 items from 10 facets grouped into four domains: physical function (8 items), psychological function (9 items), social function (8 items), and common symptoms and side effects (7 items) (14). The breast cancer-specific module consists of 3 facets and 10 items. The whole scale consists of 5 domains, 13 facets, and 42 items (15). The QLICP-BR (V2.0) has been verified in Mainland China and has good reliability, validity, and responsiveness after testing. It can be used for the determination of the quality of life in patients with breast cancer during the period of onset, treatment, and rehabilitation (15).

In order to reasonably explain the clinical significance of questionnaire measurements and scale scores, as early as 1987, Guyatt et al. proposed to use the Minimal Clinically Important Difference (MCID) as an appropriate benchmark for evaluating important changes in the responsiveness of the scale. The researchers did not define MCID and acknowledged the difficulty of quantifying MCID, indicating that the changes caused by interventions with known efficacy can provide a preliminary estimate. Two years later, Jaeschke, Singer, and Guyatt defined MCID as “the smallest change in the score of the questionnaire dimension recognized by the patient without considering side effects and costs (16).” Thus, clinicians need a systematic approach to assess the perceived benefit of a treatment based on the individual patient’s improvement in cost and risk of complications. Ideally, MCID will provide a specific threshold as a treatment target and has been widely used in this regard.

The QLICP-BR (V2.0) has been widely used in China, but MCID has not been developed, so it is not convenient for further applications. The purpose of this study is to use the QLICP-BR (V2.0) scale to develop the minimal clinically important difference (MCID) for breast cancer.

## Materials and Methods

### Subjects and Data Collection

This study was based on inpatients with a clinical breast cancer who were diagnosed by pathological examination in the Affiliated Hospital of Guangdong Medical University and the Central Hospital of Guangdong Nongken. The investigator appeared as a doctor and briefly explained the content and purpose of the investigation. After obtaining the consent of the patient and signing the informed consent form, the investigator sent the QLICP-BR (V2.0) to the patients to fill in by themselves. In total, 246 patients were included in the study. The inclusion and exclusion criteria are as follows:

Inclusion criteria: (1) Patients with a clear diagnosis, that is, those diagnosed with breast cancer by pathological examination; (2) Good reading and presentation skills, able to fill out questionnaires by themselves; (3) Volunteer to participate in the survey, no mental illness or disturbance of consciousness.

Exclusion criteria: (1) Cognitive and consciousness dysfunction; (2) Those who refuse to participate in the research or those with a low degree of cooperation; (3) At the end of life, combined with other primary cancers, other serious diseases, mental illnesses, etc.; (4) Malignant tumors that frequently metastasize.

In terms of sample size, we use the sample size calculation *formula:*


$$n=\frac{Z_{\alpha }^{2}\times pq}{d^{2}}$$

p is the effective rate of treatment, q=1-p, d represents the allowable error, and Z_α_ is the statistical quantity of the significance test. When d = 0.1p and α =0.05, $\text{n}=400\times \frac{\text{q}}{\text{p}}$ . According to experience, p =0.67,q=0.33, So n is equal to 197. In addition, according to the empirical method, the sample size should be 5-10 times that of the variable. The number of items in this scale is 42, and the sample size is suitable for 210-420 cases. The sample size of this investigation is 246 cases, which can meet the statistical requirements of sample size.

### MCIDs of the Anchor-Based Method

The anchor-based method is used to clarify the meaning of the scale’s score change by examining the relationship between the scale and the score of another independent measurement tool or other indicators. Anchor-based approaches assess the extent to which changes in measurement instruments correspond to a minimally important change defined by external indicators (17). Anchors are divided into cross-sectional anchors and longitudinal anchors (18). This article is used to compare the efficacy before and after treatment, so the longitudinal anchor was selected. First, the RS (raw score) was scored based on the number of questions contained in each domain and the patient’s options. Then, linear transformation was performed using the range method to convert the raw score into a standardized score (SS) with a value between 0 and 100. The formula for calculating the score in each domain is as follows (19):


$$\begin{array}{cc} SS=(RS-M\text{in})\times 100/R & R=M\text{ax}-Min \end{array}$$

The Q29 item “ How would you rate your overall health during the past week?” in the EORTC QLQ-C30 scale (20) was taken as the anchor and used to calculate the correlation coefficient between Q29 and the QLICP-BR (V2.0) total scale score. Then, patients who differed by one grade (criterion A) and at least one grade (criterion B) in Q29 before and after treatment were screened out. The score difference in each domain before and after treatment was calculated, and the mean of the absolute value of the difference was recorded as MCID. The calculation formula is shown in **Table 1**.

**Table 1:** Calculation formula of MCID based on anchor method.

Standard	Sample size *n*	Anchor difference *D* (Q29 Score difference)	Scale field score difference *d*	Mean ( $\bar{\left|d\right|}$ )
A: One level away	*n_1_*	*D*_1_=X_1_- X_0_=±1	*x_1_-x_0_*	$\frac{d_{1}+d_{2}+\ldots +d_{n_{1}}}{n_{1}}$
B: At least one level away	*n_2_*	*D*_2_=X_1_-X_0_=±1,2,3,4,5,6	*x_1_-x_0_*	$\frac{d_{1}+d_{2}+\ldots +d_{n_{2}}}{n_{2}}$

### MCIDs of the Distribution-Based Method

The distribution-based method uses the evaluation tool sample data distribution (variation) to determine the MCID from a statistical point of view. Commonly used indicators for calculating variation include effect size (ES), standard error of measurement (SEM), and reliability change index (RCI). The calculation formula and corresponding MCID calculation are as follows:


$$\begin{array}{cc} ES=\frac{{\bar{x}}_{1}-{\bar{x}}_{0}}{\sqrt{\sum {\left(x_{0}-{\bar{x}}_{0}\right)}^{2}/n-1}} & MCID=ES\times SD_{baseline} \end{array}$$

*X_0_* is the baseline score of the respondent;

${\bar{x}}_{0}$ is the mean baseline score of the respondent; *SD_baseline_* is the standard deviation of the baseline score of the respondent; ${\bar{x}}_{1}$ is the mean score of the respondent after treatment; and n is the sample size (21–23). In the health-related quality of life assessments, the ES is currently a relatively recognized parameter in determining the importance of group or individual changes. There is also an accepted standard for ES judgment: 0.2 is a small effect; 0.5 is a medium effect; 0.8 or greater is a large effect (23).


$$\begin{array}{cc} SEM=\sqrt{1-r}\times \sqrt{\sum {\left(x_{0}-{\bar{x}}_{0}\right)}^{2}/n-1} & MCID=X\times SD_{\text{baseline}}\times \sqrt{1-r} \end{array}$$

Where r is the reliability coefficient of the evaluation questionnaire and the test-retest reliability coefficient is generally used. If the test-retest reliability coefficient is unknown, the Cronbach coefficient can be used instead. X can be 1 (small effect), 1.96 (medium effect), or 2.77 (large effect) (22–24).


$$\begin{array}{cc} RCI=\frac{{\bar{x}}_{1}-{\bar{x}}_{0}}{\sqrt{2{\left(SEM\right)}^{2}}} & MCID=X\times SD_{baseline}\times \sqrt{2(1-\text{r)}} \end{array}$$

RCI, which is the change in questionnaire score divided by the square root of the standard measurement error (25, 26).

### Statistical Software

This survey used Epidata3.1 software to input data and SPSS 25.0 software to organize and analyze the data. The scores of various domains and the total score of the scale and the MCID value of breast cancer were calculated.

## Results

### Social-Demographic and Clinical Characteristics of Breast Cancer Patients

A total of 246 breast cancer patients were investigated in this study, all of which were women. The age distribution was between 17-77 years old. The average age was 50.07 ± 10.25. Among the patients, 5.3% were under 30 years old, 10.2% were between 30 and 40 years old, 37.0% were between 40 and 50 years old, 29.3% were between 50 and 60 years old, and 18.3% were over 60 years old. The household economy was mostly medium, accounting for 67.9% of the total population. The occupations were mostly workers and farmers, with 20 workers (8.1%) and 112 farmers (45.5.0%), respectively. The majority of patients were married, accounting for 97.2%. The most common ethnicity was Han, accounting for 97%. The distribution of educational level was mostly in Middle school or High school, accounting for 60.2%. Medical insurance was mostly used in medical forms, accounting for 91.9%. Medical treatment insurance included medical insurance for urban residents, medical insurance for urban workers, cooperative medical treatment, and commercial medical insurance. TNM stages were distributed between I-IV stages, with 53 being stage I (21.5%), 86 stage II (35.0%), 54 stage III (22.0%), and 27 stage IV (11.0%), respectively. See **Table 2** in detail.

**Table 2:** Socio-demographic and clinical characteristics of the Sample (n = 246).

Characteristics	N	%	Characteristics	N	%
**Gender**			**Marital status**		
Male	0	0	Married	239	97.2
Female	246	100	Others	7	2.8
**Age**			**Ethnicity**		
≤30	13	5.3	Han	237	96.3
30-40	25	10.2	Others	9	3.7
40-50	91	37.0	**Education**		
50-60	72	29.3	Primary school	65	26.4
≥60	45	18.3	Middle school or High school	148	60.2
			College/University	33	13.4
**Economic**			**Medical insurance**		
Poor	52	21.1	Self-paid/Private insurance	20	8.1
Fair	167	67.9	Medical insurance	226	91.9
High	27	11.0	**TNM**		
**Occupation**			I	53	21.5
Worker	20	8.1	II	86	35.0
Farmer	112	45.5	III	54	22.0
Others	114	46.3	IV	27	11.0

### MCIDs of the Anchor-Based Method

The correlation coefficient between the Q29 and QLICP-BR (V2.0) score in the EORTC QLQ-C30 scale was calculated using the Q29 item “How do you evaluate your total health condition in the past week” as an anchor, and r = 0.651 was obtained.

According to standard A, patients with a difference of one grade on Q29 before and after treatment were screened, 116 cases were effective, and the QLICP-BR (V2.0) scores in each domain and the total scale score difference before and after treatment were calculated.

In regard to socio-demographic and clinical characteristics of the sample based on Standard A, 116 (100%) patients were female, 78 (67.2%) were aged between 40 and 60 years old. Most of them had medium family income (69%) and had middle school education (64.7%). 54 (46.6%) were farmers, 100 (94.8%) were married, 112 (96.6%) were Han nationality, 107 (92.2%) had medical insurance. On clinical stages, the cases distributed in the four stages of TNM I, II, III, IV were 29, 38, 21, 14, respectively.

According to standard B, patients with at least one grade difference in Q29 before and after treatment were screened. A total of 166 patients were effective. The scores of QLICP-BR (V2.0) in each domain and the total scale score difference before and after treatment were calculated.

In terms of socio-demographic and clinical characteristics of the sample based on Standard B, all of the 166 patients were female, and 112 (67.5%) were aged between 40 and 60 years old. Most of them had medium family income (67.5%) and middle school education (64.4%). 81(48.8%) were farmers, 160 (96.4%) were married, 162 (97.6%) were Han nationality, and 155(93.4%) had medical insurance. On clinical stages, the cases distributed in the four stages of TNM I, II, III, IV were 38, 59, 31, 22, respectively.

Then, the mean and standard deviation of the difference between the scores of each domain and the total scale under the two standards were calculated and the mean of the difference as MCID was recorded. The results are shown in **Table 3**. According to standard A, the MCID values of physical domain (PHD), psychological domain (PSD), social domain (SOD), common symptoms and side effect domain (SSD), core/general module (CGD), specific domain (SPD) and the total scale (TOT) were 16.24, 11.37, 11.31, 12.07, 11.49, 10.69 and 11.23, respectively. According to standard B, the MCID values of PHD, PSD, SOD, SSD, CGD, SPD and TOT were 18.88, 15.14, 14.10, 14.50, 13.93, 12.17, and 14.23, respectively.

**Table 3:** The MCID value of QLICP-BR (V2.0) determined by anchor-based method (n_A_ = 116, n_B_ = 166).

**Domain**	**Items**	**Standard A** $\bar{x}\pm s$	**Standard B** $\bar{x}\pm s$	**Standard A MCID**	**Standard B MCID**
Physical domain (PHD)	8	16.24 ± 10.65	18.88 ± 12.31	16.24	18.88
Psychological domain (PSD)	9	11.37 ± 9.24	15.14 ± 12.51	11.37	15.14
Social domain (SOD)	8	11.31 ± 8.08	14.10 ± 13.80	11.31	14.10
Common symptoms and side effect domain (SSD)	7	12.07 ± 8.69	14.50 ± 12.08	12.07	14.50
Core/general module (CGD)	32	11.49 ± 7.41	13.93 ± 9.26	11.49	13.93
Specific domain (SPD)	10	10.69 ± 8.34	12.17 ± 9.83	10.69	12.17
Total (TOT)	42	11.23 ± 7.47	14.23 ± 9.63	11.23	14.23

### MCIDs of the Distribution-Based Methods

The distribution-based method estimates MCID based on the observed distribution of score changes. The MCID results of breast cancer were calculated using three variation indexes: ES, SEM, and RCI. We calculated the MCID results using the three indicators respectively as shown in **Tables 4**–**6**.

**Table 4:** The MCID value of QLICP-BR (V2.0) determined by ES (n = 246).

Domain	SD_baseline_	0.2ES	0.5ES	0.8ES
Physical domain (PHD)	11.42	2.28	5.71	9.14
Psychological domain (PSD)	12.92	2.58	6.46	10.34
Social domain (SOD)	10.43	2.09	5.22	8.34
Common symptoms and side effect domain (SSD)	13.17	2.63	6.56	10.54
Core/general module (CGD)	8.49	1.70	4.25	6.79
Specific domain (SPD)	12.16	2.43	6.08	9.73
Total (TOT)	8.70	1.74	4.35	6.96

**Table 5:** The MCID value of QLICP-BR (V2.0) determined by SEM (n = 246).

Domain	r	SD_baseline_	SEM	1.96SEM
Physical domain (PHD)	0.86	11.42	4.27	8.38
Psychological domain (PSD)	0.81	12.92	5.63	11.04
Social domain (SOD)	0.82	10.43	4.43	8.67
Common symptoms and side effect domain (SSD)	0.85	13.17	5.10	10.00
Core/general module (CGD)	0.80	8.49	3.80	7.44
Specific domain (SPD)	0.83	12.16	5.01	9.83
Total (TOT)	0.79	8.70	3.99	7.81

**Table 6:** The MCID value of QLICP-BR (V2.0) determined by RCI (n = 246).

Domain	r	SD_baseline_	RCI	1.96RCI	2.77RCI
Physical domain (PHD)	0.86	11.42	6.04	11.84	16.74
Psychological domain (PSD)	0.81	12.92	7.96	15.61	22.06
Social domain (SOD)	0.82	10.43	6.26	12.27	17.33
Common symptoms and side effect domain (SSD)	0.85	13.17	7.21	14.14	19.98
Core/general module (CGD)	0.80	8.49	5.37	10.52	14.87
Specific domain (SPD)	0.83	12.16	7.09	13.90	19.64
Total (TOT)	0.79	8.70	5.64	11.05	15.62

As can be seen from **Table 4**, when ES = 0.2, the MCID values for each domain and the total scale were 2.28, 2.58, 2.09, 2.63, 1.70, 2.43 and 1.74, respectively. When ES = 0.5, the MCID values of each domain and the total scale were 5.71, 6.46, 5.22, 6.56, 4.25, 6.08 and 4.35, respectively. When ES = 0.8, the MCID values of each domain and the total scale were 9.14, 10.34, 8.34, 10.54, 6.79, 9.73 and 6.96, respectively.

The MCIDs calculated for above domains/the total when SEM was used as an intermediary index were 4.27, 5.63, 4.43, 5.10, 3.80, 5.01 and 3.99, respectively. The MCIDs calculated for above domains/the total when 1.96SEM was used as the intermediary index were 8.38, 11.04, 8.67, 10.00, 7.44, 9.83 and 7.81, respectively.

The MCIDs calculated for above domains/the total when RCI was used as the intermediary index were 6.04, 7.96, 6.26, 7.21, 5.37, 7.09 and 5.64, respectively. The MCIDs calculated for above domains/the total when 1.96RCI was used as the intermediary index were 11.84, 15.61, 12.27, 14.14, 10.52, 13.90 and 11.05, respectively. The MCIDs calculated for above domains/the total when 2.77RCI was used as the intermediary index were 16.74, 22.06, 17.33, 19.98, 14.87, 19.64, and 15.62, respectively.

## Discussions

MCID is a difference score that is sufficient to reflect the effect on patients after clinical treatment, and its main function is to help clinical and research personnel to determine whether changes in the score of the scale have clinical significance. Clearly, clinicians also need a systematic way to assess the perceived benefit of a certain treatment based on individual patient improvement relative to both cost and risk of complications (27). At the same time, it can also further explain the score of the scale, so as to provide a scientific basis for clinical and scientific researchers to deal with patients of different degrees more specifically (28). Therefore, it is critical that the MCID score is a valid and stable measure. A low MCID may result in overestimating the positive effects of treatment, whereas a high MCID value may incorrectly classify patients as failing to respond to treatment when in fact the treatment was beneficial (26).

In clinical studies, a lot of methodological approaches have been reported to calculate MCID such as the anchor-based method, distribution-based method, expert opinion method, literature analysis method, etc (29). In general, methodological approaches can be classified into two broad groups: anchor-based and distribution-based (29–32). The two kinds of methods can get a variety of results from different angles, which is conducive to comparison and user selection according to the situation. But each method has its advantages and disadvantages. An anchor-based approach relies on external calibration, which must be easy to interpret and strong in relation to the quality of life. But choosing different anchors will result in different MCID values. The distribution-based method takes into account the measurement error and also has a specific calculation formula, which is easy to implement, but easily affected by sample size (33, 34). Therefore, most scholars recommend applying multiple methods where the anchoring method is used as the main method and the distribution method is used as a supplement to determine MCID.

The EORTC-QLQ-C30 is a 30-item health-related quality of life questionnaire for cancer patients. Q30(How would you rate your overall quality of life during the past week)? and Q29 (How would you rate your overall health during the past week)? in the scale are comprehensive items and are often used as anchors. We calculated the correlation coefficients of Q29, Q30 and QLICP-BR (V2.0) scales respectively. The correlation coefficients were 0.651 and 0.588, respectively. Obviously, the correlation coefficient between Q29 and scale score is larger. At the same time, the content of Q29 is easier to be understood by patients. Therefore, we choose Q29 as the anchor. In this study, using the anchor-based method, according to standard A, the MCIDs of PHD, PSD, SOD, SSD, CGD, SPD and TOT were 16.24, 11.37, 11.31, 12.07, 11.49, 10.69 and 11.23, respectively; ranging from 10.69 to 16.24 points. According to standard B, the MCIDs of PHD, PSD, SOD, SSD, CGD, SPD and TOT were 18.88, 15.14, 14.10, 14.50, 13.93, 12.17 and 14.23, respectively, ranging from 12.17 to 18.88 points. It can be seen that according to criterion A, patients are more likely to obtain meaningful clinical change values; in other words, the quality of life of breast cancer patients is more likely to be judged as improved.

However, the selected anchor Q29 and the total scale score correlation coefficient r was 0.651, which is not too high and may affect the reliability of MCID. In addition, the selected anchor is relatively single, which may cause unstable results. So, we continued to use the distribution-based method and calculated the MCID under the three variation indicators of ES, SEM, and RCI.

In the distribution-based MCID study, When ES = 0.2, ES = 0.5, and ES = 0.8 were used as intermediary indicators, the MCID values of the scores for each domain and the overall scale were ranged from 1.70 to 2.63 points, 4.25 to 6.56 points, and 6.79 to 10.54 points, respectively. The MCIDs of the scores for each domain and the overall scale calculated with SEM and 1.96SEM as the intermediary indicators were ranged from 3.80 to 5.63 points and 7.44 to 11.04 points, respectively. When RCI, 1.96RCI, and 2.77RCI were used as the intermediary index, the calculated MCIDs of the scores for each domain and the overall scale were ranged from 5.37 to 7.96 points, 10.52 to 15.61 points, and 14.87 to 22.06 points, respectively.

In the evaluation of clinical efficacy, the clinicians and researchers can judge clinical significance by these MCIDs. Taking 0.5ES method as an example, the overall QOL of patients can be evaluated as clinical significance when the QOL change before and after treatment is over 4.35 points for its MCID is 4.35. Similarly, the MCID of the MFSI-SF identified by Chan A et al. ranged from 4.50 to 10.79 points, and can be used to interpret the clinical significance of fatigue deterioration in patients with breast cancer (35). Quinten C et al. evaluated the short and long-term effects of chemotherapy on the reported quality of life (QOL) and patient-clinician symptom reporting in older breast cancer patients with an MCID of 10. Results showed that symptom burden and diminished QOL in an older breast cancer population receiving adjuvant TC chemotherapy are short-lived and disappear after a while with no long-term differences compared to a similar population not receiving chemotherapy (36).

In this study, anchor method and distribution method were used to calculate MCID of breast cancer. When the MCID calculated by anchor method is smaller than that calculated by distribution method or the correlation coefficient between the selected anchor and the measured scale is small (the absolute value r at least 0.5 is currently recommended), the results of the distribution method are recommended as the MCIDs of breast cancer (37). In the calculation of MCID, the anchor method is generally preferred. However, the distribution method is also considered comprehensively if there is no good anchor or the number of patients is not large.

In summary, the MCID value calculated by the two standards of the anchor method was similar to 0.8ES, 1.96SEM, and 1.96RCI. Therefore, these indicators should be given priority in the development of MCID for breast cancer.

Similar results have also been obtained in developing MCID for breast cancer in foreign countries. Alexandre Chan et al. used the anchor-based method, distribution-based method, and ROC curve method to develop the MCID of breast cancer, and the results showed that the MCID of the MFSI-SF identified by all ranged from 4.50 to 10.79 points (38). Yin Ting Cheung et al. used the anchor-based method, distribution-based method, and ROC curve method to develop MCID for breast cancer, and the results were as follows: the estimates of 6.9-10.6 points as MCID can facilitate the interpretation of patient-reported cognitive weakness and sample size in future studies (39). These results are similar to the result of our study, but the MCID value of breast cancer we developed was relatively higher than that of the foreign results. This may be caused by the use of different scales whose scores ranging from 0 to 100 and cultural differences between China and foreign countries.

From the results obtained, we conclude that the MCID value calculated by the two standards of the anchor-based method was similar to 0.8ES, 1.96SEM, and 1.96RCI of the distribution-based methods. Therefore, when selecting the minimal clinical important difference for breast cancer patients, the results of the anchor-based method can be used preferentially, also 0.8ES, 1.96SEM, 1.96RCI of distribution-based methods can be used.

Of course, this study has certain limitations. The methods used in this study include the anchor-based method and the distribution-based method. Other methods can also be used to formulate MCID such as the ROC curve method and the response cumulative distribution function method. Additionally, the sample size of this subject research can be expanded to make the MCID more stable. In future studies, the sample size of breast cancer patients will be expanded, and multiple methods will be used to jointly develop MCID for breast cancer, so as to ensure more stable results.

## Data Availability Statement

The raw data supporting the conclusions of this article will be made available by the authors, without undue reservation.

## Ethics Statement

The study protocol and the informed consent form were approved by the IRB (Institutional Review Board) of the affiliated hospital of Guangdong Medical University (PJ2012052, YJYS2019010). The patients/participants provided their written informed consent to participate in this study. Written informed consent was obtained from the individual(s) for the publication of any potentially identifiable images or data included in this article.

## Author Contributions

CW designed the study. FL, JZ, and HC performed the data collection. FL,JT, and YL performed data analyses and drafted the manuscript. CW revised the manuscript deeply. All authors contributed to interpreting the data, and have read and approved the final manuscript.

## Funding

This study is supported by the National Natural Science Foundation of China (71974040, 81273185), Dongguan Science and Technology of Social Development Program (20211800905102).

## Conflict of Interest

The authors declare that the research was conducted in the absence of any commercial or financial relationships that could be construed as a potential conflict of interest.

## Publisher’s Note

All claims expressed in this article are solely those of the authors and do not necessarily represent those of their affiliated organizations, or those of the publisher, the editors and the reviewers. Any product that may be evaluated in this article, or claim that may be made by its manufacturer, is not guaranteed or endorsed by the publisher.

## Abbreviations

MCID, minimal clinically important differences; QLICP-BR, Quality of Life Instruments system in breast cancer patients; EORTC, European Organization for Research and Treatment; ES, effect size; SEM, standard error of measurement; RCI, reliability change index; PHD, physical domain; PSD, psychological domain; SOD, social domain; SSD, common symptoms and side effect domain; CGD, core/general module; SPD, specific domain; TOT, total; HRQOL, health-related quality of life; ROC curve, receiver operating characteristic.
