# Supplementary material for: Establishing Minimal Clinically Important Differences for the Quality of Life Instrument in Patients With Breast Cancer QLICP-BR (V2.0) Based on Anchor-Based and Distribution-Based Methods
Source: Front Oncol. 2022 May 2;12:753729. doi: 10.3389/fonc.2022.753729 (PMC9108929; doi:10.3389/fonc.2022.753729)
Supplement: Supplementary file 1 [file DataSheet_1.doc]

**[Appendix](https://fanyi.so.com/?src=onebox#appendix) I**

**Quality of Life Instruments for Cancer Patients – Breast cancer Scale**

**QLICP-BR (V2.0)**

***INSTRUCTION:*** This questionnaire helps doctors know your feeling about your health condition in the past week. Your answers will help them choose the appropriate treatment and rehabilitation strategy. There is no right or wrong answer. Please read the following questions carefully, and circle the number most relevant how your feeling. You may choose the answer closest to your true feeling in case you are not sure how to answer the question. The information that you provide will remain strictly confidential.

**Physical Function**

|  |  | **Not at all** | **A little** | **Moderately** | **Very much** | **Extremely** |
| --- | --- | --- | --- | --- | --- | --- |
| GPH1 | Have you had a good appetite? | 1 | 2 | 3 | 4 | 5 |
| GPH2 | Were you satisfied with your sleep? | 1 | 2 | 3 | 4 | 5 |
| GPH3 | Has the disease or treatment affected your sexual activities？ | 1 | 2 | 3 | 4 | 5 |
| GPH4 | Were your bowels acting properly? | 1 | 2 | 3 | 4 | 5 |
| GPH5 | Could you work? (e.g., doing housework, working, washing or farming, etc）? | 1 | 2 | 3 | 4 | 5 |
| GPH6 | Could you take care of your daily life? (e.g., eating, dressing, washing, using toilet）? | 1 | 2 | 3 | 4 | 5 |
| GPH7 | Did you have any trouble going up and down stairs？ | 1 | 2 | 3 | 4 | 5 |
| GPH8 | Were your urine passing normally? | 1 | 2 | 3 | 4 | 5 |

**Psychological Function**

|  |  | **Not at all** | **A little** | **Moderately** | **Very much** | **Extremely** |
| --- | --- | --- | --- | --- | --- | --- |
| GPS1 | Did you feel depressed or sad? | 1 | 2 | 3 | 4 | 5 |
| GPS2 | Did you find life enjoyable? | 1 | 2 | 3 | 4 | 5 |
| GPS3 | Have you felt fretful or irritable？ | 1 | 2 | 3 | 4 | 5 |
| GPS4 | Has the disease caused your memory to decline? | 1 | 2 | 3 | 4 | 5 |
| GPS5 | Were you worried about your health getting worse? | 1 | 2 | 3 | 4 | 5 |
| GPS6 | Could you treat the illness positively and optimistically? | 1 | 2 | 3 | 4 | 5 |
| GPS7 | Were you confident in overcoming the disease? | 1 | 2 | 3 | 4 | 5 |
| GPS8 | Were you afraid of your illness | 1 | 2 | 3 | 4 | 5 |
| GPS9 | Has your concentration been affected by the disease? | 1 | 2 | 3 | 4 | 5 |

**Social Function**

|  |  | **Not at all** | **A little** | **Moderately** | **Very much** | **Extremely** |
| --- | --- | --- | --- | --- | --- | --- |
| GSO1 | Could you get care and support from your family? | 1 | 2 | 3 | 4 | 5 |
| GSO2 | Could you get the care and support from your friends and relatives? | 1 | 2 | 3 | 4 | 5 |
| GSO3 | Could you undertake appropriate family roles (such as parent, husband, wife)? | 1 | 2 | 3 | 4 | 5 |
| GSO4 | Did you turn to others for help when you are in trouble? | 1 | 2 | 3 | 4 | 5 |
| GSO5 | Have economic problems caused by your illness or treatment affected your life? | 1 | 2 | 3 | 4 | 5 |
| GSO6 | Has the disease or treatments interfered with your work or housework? | 1 | 2 | 3 | 4 | 5 |
| GSO7 | Have you had good relations with your families? | 1 | 2 | 3 | 4 | 5 |
| GSO8 | Have your illness and treatment prevented you from interacting with others? | 1 | 2 | 3 | 4 | 5 |

**Common symptoms and side-effects domain (SSD)**

|  |  | **Not at all** | **A little** | **Moderately** | **Very much** | **Extremely** |
| --- | --- | --- | --- | --- | --- | --- |
| GSS1 | Did you have any nausea or vomiting? | 1 | 2 | 3 | 4 | 5 |
| GSS2 | Did you have hair loss? | 1 | 2 | 3 | 4 | 5 |
| GSS3 | Did you have mouth ulcers? | 1 | 2 | 3 | 4 | 5 |
| GSS4 | Did you have pain? | 1 | 2 | 3 | 4 | 5 |
| GSS5 | Were you losing weight? | 1 | 2 | 3 | 4 | 5 |
| GSS6 | Did you have a bitter or dry mouth? | 1 | 2 | 3 | 4 | 5 |
| GSS7 | Have you felt fatigue easily? | 1 | 2 | 3 | 4 | 5 |

**Specific Module**

|  |  | **Not at all** | **A little** | **Moderately** | **Very much** | **Extremely** |
| --- | --- | --- | --- | --- | --- | --- |
| SBR1 | Did you feel swelling and pain on the affected side or contralateral breast? | 1 | 2 | 3 | 4 | 5 |
| SBR2 | Were your upper limbs restricted? | 1 | 2 | 3 | 4 | 5 |
| SBR3 | Did you feel swelling or pain in the upper limbs on the affected side | 1 | 2 | 3 | 4 | 5 |
| SBR4 | Were there any abnormal changes in your breast skin? | 1 | 2 | 3 | 4 | 5 |
| SBR5 | Did you have any abnormal lumps on the breast or armpit on your affected or contralateral side? | 1 | 2 | 3 | 4 | 5 |
| SBR6 | Did you care about changes in your body image? | 1 | 2 | 3 | 4 | 5 |
| SBR7 | Did you think it will have a big impact on the couple's life (sex life) after the illness? | 1 | 2 | 3 | 4 | 5 |
| SBR8 | Did symptoms such as flushing, muscle and joint pain, and fatigue appear again after treatment? | 1 | 2 | 3 | 4 | 5 |
| SBR9 | Did you have chest tightness or shortness of breath? | 1 | 2 | 3 | 4 | 5 |
| SBR10 | Did you have bone pain (such as ribs, spine, pelvis, etc.)? | 1 | 2 | 3 | 4 | 5 |

**[Appendix](https://fanyi.so.com/?src=onebox#appendix) II:**

**QLICP-BR (V2.0) adopted five-point isometric scoring method, which was successively counted as 1, 2, 3, 4 and 5 points. There are positive and negative items in the scale. The higher the score of the positive item, the better the quality of life; the higher the score of the reverse item, the worse the quality of life. For positive items, there is no need to transform, and the original score is the item score. For reverse items, forward transformation is required, as follows:**

**Positive item score= (0+ answer option number)**

**Reverse item score=(6- answer option number)**

**Table 1 The construct and scoring method of QLICP-BR (V2.0)**

| Domains/facets | No. of items | Range scores | Scoring method | |
| --- | --- | --- | --- | --- |
|  |  |  | RS | SS |
| **Physical domain (PHD)** | **8** | **8-40** | **BPF + CMA** | **(RS-8)×100/32** |
| Basic physiologic function (BPF) | 5 | 5-25 | GPH1 + GPH2 + GPH3 + GPH4 + GPH8 | (RS-5)×100/20 |
| Capacity of movement and activity (CMA) | 3 | 3-15 | GPH5 + GPH6 + GPH7 | (RS-3)×100/12 |
| **Psychological domain (PSD)** | **9** | **9-45** | **COG + EMO + WIL** | **(RS-9)×100/36** |
| Cognition (COG) | 2 | 2-10 | GPS4+ GPS9 | (RS-2)×100/8 |
| Emotion (EMO) | 5 | 5-25 | GPS1+ GPS2+ GPS3 + GPS5+ GPS8 | (RS-5)×100/20 |
| Will (WIL) | 2 | 2-10 | GPS6 + GPS7 | (RS-2)×100/8 |
| **Social domain (SOD)** | **8** | **8-40** | **INC + SSS + SOR** | **(RS-8)×100/32** |
| Interpersonal communication (INC) | 2 | 2-10 | GSO7 + GSO8 | (RS-2)×100/8 |
| Social support and safety (SSS) | 4 | 4-20 | GSO1+ GSO2+ GSO4 + GSO5 | (RS-4)×100/16 |
| Social role (SOR) | 2 | 2-10 | GSO3 + GSO6 | (RS-2)×100/8 |
| **Common symptoms and side-effects domain (SSD)** | **7** | **7-35** | **CST + CSE** | **(RS-7)×100/28** |
| Common symptoms (CST) | 3 | 3-15 | GSS4+ GSS5+ GSS7 | (RS-3)×100/12 |
| Common side-effects (CSE) | 4 | 4-20 | GSS1+ GSS2+ GSS3+ GSS6 | (RS-4)×100/16 |
| **Core/general domain (CGD)*** | **32** | **32-160** | **PHD + PSD + SOD + SSD** | **(RS-32)×100/128** |
| **Specific domain (SPD)** | **10** | **10-50** | **CLS + TSE+ SPE** | **(RS-10)×100/40** |
| Clinical symptoms (CLS) | 5 | 5-25 | SBR1 + SBR2+SBR3+SBR4+SBR5 | (RS-5)×100/20 |
| Therapeutic side-effects (TSE) | 3 | 3-15 | SBR8+SBR9+SBR10 | (RS-3)×100/12 |
| Specific psychological effects (SPE) | 2 | 2-10 | SBR6 + SBR7 | (RS-2)×100/8 |
| **Total (TOT)** | **42** | **42-210** | **PHD + PSD + SOD + SSD + SPD** | **(RS-42)×100/168** |

RS: raw score, SS: standardized score.
